# A Narrative Review on Nitrate-Rich Diets as Adjuncts to Antihypertensive Therapy: Enhancing Treatment Efficacy via Oxidative Stress Modulation

**DOI:** 10.3390/biomedicines14010039

**Published:** 2025-12-23

**Authors:** Mila Silva-Cunha, Carla Speroni Ceron, Heitor Moreno, José Eduardo Tanus-Santos

**Affiliations:** 1 Department of Pharmacology, Ribeirao Preto Medical School, University of Sao Paulo, Ribeirao Preto 14049-900, Brazil; milacunha@usp.br; 2 Department of Biological Sciences, Institute of Exact and Biological Sciences, Federal University of Ouro Preto, Ouro Preto 35402-136, Brazil; carla.ceron@ufop.edu.br; 3 Department of Internal Medicine, State University of Campinas, Campinas 13083-894, Brazil; hmoreno@uol.com.br

**Keywords:** nitric oxide signaling, endothelial dysfunction, redox homeostasis, antioxidant-rich diet

## Abstract

Arterial hypertension (AH) is a highly prevalent, multifactorial cardiovascular condition characterized by endothelial dysfunction, increased oxidative stress, and impaired nitric oxide (NO) bioavailability. While pharmacological treatment is primarily directed toward blood pressure reduction, accumulating evidence indicates that several antihypertensive drug classes also confer antioxidant and vasculoprotective benefits. Concurrently, dietary intake of inorganic nitrate and nitrite has gained attention as an adjunctive approach to restore NO signaling and redox homeostasis. This narrative review summarizes current evidence regarding the antioxidant effects of major antihypertensive drug classes and examines the contribution of nitrate- and nitrite-rich diets to the modulation of oxidative stress and vascular dysfunction in AH. A systematic search of PubMed, EMBASE, Scopus, ScienceDirect, Web of Science, Google Scholar, and Food and Drug Administration (FDA) databases was performed for studies published between August and December 2025. Experimental and clinical investigations assessing oxidative stress markers, endothelial function, or NO-related outcomes in AH were selected following title and abstract screening and full-text evaluation. Available data indicate that angiotensin-converting enzyme inhibitors, angiotensin II receptor blockers, diuretics, β-blockers, and calcium channel blockers mitigate oxidative stress via mechanisms including NADPH oxidase suppression, decreased reactive oxygen species production, reinforcement of endogenous antioxidant systems, and restoration of endothelial NO bioavailability. Moreover, dietary nitrate and nitrite support vascular function through activation of the nitrate–nitrite–NO pathway. Combining nitrate- and antioxidant-rich dietary strategies with antihypertensive agents that lack inherent redox-modulating activity may enhance blood pressure control and lower cardiovascular risk. Nevertheless, well-designed long-term randomized clinical trials are needed to elucidate class-specific interactions and underlying redox mechanisms.

## 1. Introduction

Arterial hypertension (AH) is one of the most prevalent clinical conditions worldwide and represents a major risk factor for severe cardiovascular (CV) events, including acute myocardial infarction, stroke, and premature death [1]. According to the World Health Organization (WHO), more than 1.28 billion people are estimated to be living with hypertension, a condition responsible for millions of deaths each year [2]. Essential hypertension, the most common form of the disease, has no clearly defined etiology and is often silent and asymptomatic until serious complications arise; therefore, early diagnosis and adequate control are critical [3].

The management of hypertension remains a clinical challenge and typically requires a combination of lifestyle modifications [4] and pharmacological interventions [5]. Approximately 60% of hypertensive patients need two or more antihypertensive agents from different pharmacological classes to achieve optimal blood pressure (BP) control [6]. Nevertheless, a substantial proportion of these individuals continue to exhibit BP levels above recommended targets, even under continuous therapy [7].

The difficulty in achieving sustained BP control may be attributed to multiple factors, including poor treatment adherence, the presence of comorbidities, individual genetic variability, drug resistance, and, notably, the complexity of the pathophysiological mechanisms underlying AH [8,9]. This highlights the importance of therapeutic approaches that address not only BP reduction but also the biochemical and molecular processes contributing to disease development and progression.

Among the key pathophysiological mechanisms associated with hypertension are oxidative stress [10] and nitric oxide (NO) deficiency [11]. NO is a signaling molecule synthesized by endothelial cells via endothelial nitric oxide synthase (eNOS) and plays a fundamental role in the regulation of vascular tone. Its vasodilatory effects, along with the inhibition of platelet aggregation and prevention of leukocyte adhesion to the endothelium, confers upon NO a central role in maintaining CV homeostasis [12].

Some antihypertensive agents display antioxidant effects in addition to their primary pharmacological actions. The β-blocker nebivolol, for example, has been shown to reduce reactive oxygen species (ROS) levels in cardiac tissue in experimental models of hypertension [13]. Similarly, spironolactone and hydrochlorothiazide, widely used as first-line antihypertensive drugs, have demonstrated the ability to decrease NADPH oxidase-mediated superoxide anion (O_2_^−^) production [14]. The reaction between NO and superoxide results in the formation of peroxynitrite (ONOO^−^), a highly reactive oxidant that further reduces NO bioavailability and limits its cardioprotective effects [15].

In this context, diets rich in inorganic nitrate (NO_3_^−^) and nitrite (NO_2_^−^), which act as alternative NO donors [16], may be beneficial for hypertensive patients whose pharmacological treatment does not include agents with antioxidant properties. In addition to restoring tissue NO levels these anions exhibit antioxidant activity by reducing superoxide concentrations and mitigating ROS-induced cellular damage [17,18,19].

Therefore, this study aims to review the available scientific literature and compile evidence regarding the antioxidant effects of drugs commonly used in the therapy of AH. Furthermore, it discusses the potential benefits of inorganic nitrate and nitrite supplementation as an adjunctive strategy, particularly in therapeutic regimens that do not include agents with intrinsic antioxidant activity. By integrating these aspects, this review seeks to expand the understanding of more effective therapeutic approaches that consider not only hemodynamic control but also the molecular mechanisms underlying hypertension pathophysiology.

## 2. Methods

For the development of this narrative review, a comprehensive process involving study identification, eligibility assessment, selection, and manuscript preparation was conducted. The literature search was carried out using PubMed, EMBASE, Scopus, ScienceDirect, Web of Science, Google Scholar, and databases provided by the regulatory agency Food and Drug Administration (FDA). Searches were performed between August and December 2025 and included the keyword “oxidative stress” in combination with terms such as hypertension, endothelial dysfunction, mitochondrial dysfunction, antihypertensive drugs, angiotensin-converting enzyme inhibitors, angiotensin II receptor blockers, diuretics, β-blockers, calcium channel blockers, centrally acting antihypertensive agents, dietary nitrate, inorganic nitrite, NO bioavailability, nitrate-rich diet, and cardiovascular risk.

Study eligibility was evaluated in two stages. First, titles and abstracts were screened to ensure relevance to the subject matter. Articles selected from each search were organized using the reference management software EndNote Basic Web™. In the second stage, full-text articles were reviewed and duplicate records were excluded. Studies that aligned with the objectives of this review were included and synthesized to construct the final narrative. As this is a narrative review, protocol registration on specialized platforms was not required [20].

## 3. Oxidative Stress and Endothelial Dysfunction in Hypertension

**Figure 1:**
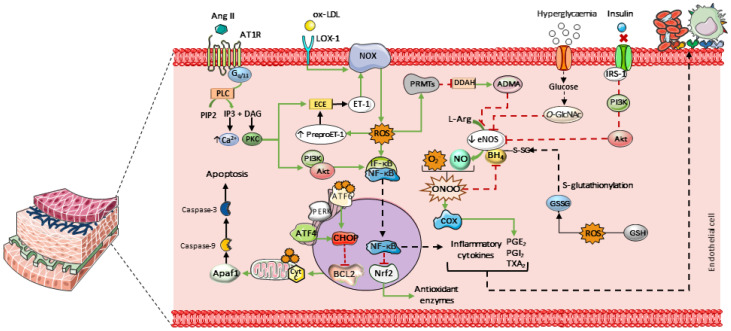
Main mechanisms contributing to endothelial dysfunction. Elevated levels of LDL and endothelin-1 (ET-1) promote cellular oxidative stress by activating NADPH oxidase (NOX), leading to increased intracellular ROS. Hyperglycemia enhances the formation of O-linked N-acetylglucosamine (O-GlcNAc), which competes with eNOS for phosphorylation. In insulin-resistant states, mutations in the gene encoding insulin receptor substrate-1 (IRS-1) impair its ability to phosphorylate eNOS via the PI3K/Akt signaling pathway, resulting in reduced eNOS activation and NO production. Parts of the figure were drawn using pictures from Servier Medical Art. Servier Medical Art by Servier is licensed under a Creative Commons Attribution 4.0 unported license.

Endothelial dysfunction is characterized by NO deficiency, resulting from either reduced NO production or increased NO inactivation. This condition is favored by factors such as elevated circulating levels of low-density lipoproteins (LDL), commonly observed in dyslipidemia. These alterations promote cellular oxidative stress and activate NADPH oxidase via the lectin-like oxidized LDL receptor-1 (LOX-1) in response to oxidized LDL (LDL-ox) [21,22]. Endothelin-1 (ET-1) also contributes to NADPH oxidase activation. Furthermore, NADPH oxidase activity is stimulated through activation of the angiotensin II (Ang II) type 1 receptor (AT_1_R), which is upregulated by increased activity of the renin–angiotensin–aldosterone system (RAAS), as observed in hypertension (Figure 1) [23,24].

Peroxynitrite is formed through the reaction between NO and superoxide. This highly reactive species stimulates cyclooxygenase-1 and 2 (COX-1 and COX-2), leading to increased synthesis of prostaglandins and thromboxanes (PGE_2_, PGI_2_, and TXA_2_, respectively). Together with inflammatory cytokines, these mediators enhance leukocyte adhesion to the endothelium and promote platelet aggregation [25,26,27]. In addition, peroxynitrite oxidizes tetrahydrobiopterin (BH_4_), an essential eNOS cofactor, leading to enzyme uncoupling. Similarly, S-glutathionylation of BH_4_ induces conformational changes that impair its interaction with eNOS. This process is primarily driven by glutathione disulfide (GSSG), whose formation is favored by ROS-mediated oxidation of reduced glutathione (GSH) [28].

These reactive species also stimulate the expression of protein arginine methyltransferases (PRMTs), which suppress the activity of dimethylarginine dimethylaminohydrolase (DDAH). Inhibition of DDAH leads to the accumulation of asymmetric dimethylarginine (ADMA), an endogenous inhibitor of eNOS [29]. Additionally, hyperglycemia enhances the formation of O-linked N-acetylglucosamine (O-GlcNAc) in vascular tissue. O-GlcNAcylation competes with phosphorylation sites on NOS, thereby impairing NO-dependent vasodilation [30]. In insulin-resistant states, mutations affecting insulin receptor substrate-1 (IRS-1) compromise its ability to activate eNOS through the phosphoinositide 3-kinase (PI3K)/Akt pathway. As a result, this signaling route is attenuated, contributing to reduced endothelial NO production and bioavailability—hallmarks of several cardiovascular diseases (Figure 1) [31,32].

Elevated ROS levels also favor leukocyte adhesion by activating the inhibitor of κB (IκB) kinase complex, which induces phosphorylation and dissociation of the IκB–nuclear factor κB (NF-κB) complex. Once translocated to the nucleus, NF-κB promotes the transcription of pro-inflammatory genes, including tumor necrosis factor-α (TNF-α), interleukins 1 and 8, E-selectin, and adhesion molecules such as vascular cell adhesion molecule-1 (VCAM-1) and intercellular adhesion molecule-1 (ICAM-1) in vascular endothelial cells [33,34]. In addition, NF-κB exerts an inhibitory effect on nuclear factor erythroid 2-related factor 2 (Nrf2), leading to decreased expression of antioxidant enzymes such as GSH, catalase (CAT), and superoxide dismutase (SOD) [35].

Endothelial cell apoptosis represents a potential outcome of this oxidative environment. Intracellular ROS alter the activity of endoplasmic reticulum stress sensors, such as activating transcription factor 6 (ATF6) and protein kinase RNA-like endoplasmic reticulum kinase (PERK). PERK regulates the expression of activating transcription factor 4 (ATF4), and elevated levels of ATF4, together with ATF6, are linked to increased expression of C/EBP homologous protein (CHOP), a pro-apoptotic transcription factor that translocates to the nucleus [36,37]. CHOP promotes apoptosis by inhibiting Bcl-2, a key regulator of anti-apoptotic proteins [38]. These proteins normally act at the mitochondrial level to prevent cytochrome c (cyt c) release. Once released, cyt c facilitates apoptosome formation via Apaf-1 and triggers caspase-9 activation, which subsequently activates the effector caspase-3 (Figure 1) [39].

Altered activity of mitochondrial electron transport chain complexes I and III enhances electron leakage and excessive production of mitochondrial ROS (mtROS), particularly superoxide anions [40]. Concurrently, mitochondrial oxidative stress induces mitochondrial DNA (mtDNA) damage, impairs oxidative phosphorylation, and reduces mitochondrial biogenesis. These effects are associated with decreased expression of mitochondrial transcription factor A (TFAM), a key regulator of mtDNA replication, maintenance, and transcription [41]. Disruption of mtDNA homeostasis compromises the expression of mitochondrial genes encoding respiratory chain subunits. Collectively, these alterations contribute to pro-inflammatory signaling and endothelial energy metabolism dysfunction [42].

As a consequence, the endothelium progressively loses its physiological functions, initiating a cascade of inflammatory and pro-apoptotic events that compromise vascular integrity. The resulting loss of endothelial homeostasis promotes sustained vasoconstriction, chronic inflammation, and vascular remodeling, thereby exacerbating endothelial injury and increasing susceptibility to thrombosis and atherosclerotic plaque formation [43]. Accordingly, improving NO bioavailability and attenuating oxidative stress emerge as key therapeutic targets in antihypertensive treatment, which are essential for preventing severe cardiovascular outcomes.

## 4. Complexity and Effectiveness of Antihypertensive Treatment

According to the American Guideline for the Prevention, Detection, Evaluation, and Treatment of Hypertension, updated in 2025, AH is a multifactorial disorder arising from complex interactions among genetic, environmental, and behavioral factors. Within this framework, CV risk stratification enables the individualization of therapeutic decisions and the establishment of treatment targets to be achieved through appropriate antihypertensive therapy and lifestyle interventions [44].

Nevertheless, an analysis published by Zhou et al. (2021) reported that the global BP control rate among treated patients is only approximately 20% [45]. When monotherapy fails to achieve adequate BP reduction, clinicians may consider drug substitution, dose escalation, or the addition of other antihypertensive agents to the therapeutic regimen [44]. Even with combination therapy, BP control may remain suboptimal, particularly in patients with comorbid conditions, underscoring the ongoing challenges in hypertension management [46,47,48].

Furthermore, despite adequate BP control, many patients continue to exhibit endothelial dysfunction and elevated levels of ROS, which sustain reduced NO bioavailability and vascular inflammation [49,50,51]. This pro-oxidative environment promotes vascular remodeling [52] and contributes to the progression of target-organ damage affecting the heart, kidneys, and brain [53,54]. Consequently, therapeutic strategies focused exclusively on BP lowering may be insufficient to fully reverse underlying vascular abnormalities.

In this context, certain antihypertensive drugs have demonstrated beneficial effects beyond BP reduction. These agents differ in their molecular characteristics and mechanisms of action. By suppressing RAAS activity, some antihypertensives indirectly reduce oxidative stress [55,56]. Others possess thiol groups within their chemical structure, conferring intrinsic antioxidant properties [57], as discussed below.

## 5. Antioxidant Properties of Antihypertensive Drugs

### 5.1. Angiotensin-Converting Enzyme Inhibitors

Angiotensin-converting enzyme inhibitors (ACEIs) are a class of antihypertensive agents that act by inhibiting the cleavage of the C-terminal dipeptide from angiotensin I and bradykinin, leading to reduced activity of the RAAS and activation of the kallikrein–kinin pathway. Through this mechanism, ACEIs decrease the formation and vasoconstrictor effects of Ang II while enhancing the vasodilatory and vasculoprotective actions mediated by bradykinin [58,59]. In addition, reduced Ang II levels result in lower aldosterone secretion, thereby decreasing sodium and water retention, attenuating sympathetic nervous system activity, and contributing to the prevention of cardiac remodeling [60].

ACEIs approved by the FDA include benazepril, captopril, enalapril, fosinopril, lisinopril, moexipril, perindopril, quinapril, ramipril, and trandolapril [61]. Notably, captopril contains a thiol group (-SH) in its structure, which provides electron-donating capacity and enables the scavenging of ROS, including hydrogen peroxide (H_2_O_2_) and singlet oxygen (^1^O_2_), as demonstrated in in vitro studies (Table 1) [57,62].

**Figure 2:**
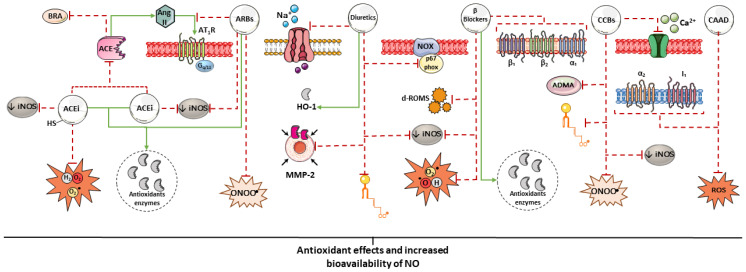
Summary of some mechanisms potentially associated with the antioxidant effects of antihypertensive drugs. The antioxidant potential of antihypertensive agents may arise from the direct scavenging of free radicals, stimulation of antioxidant enzyme expression, and indirectly from the reduction of blood pressure (BP). Parts of the figure were drawn using pictures from Servier Medical Art. Servier Medical Art by Servier is licensed under a Creative Commons Attribution 4.0 unported license.

In contrast, such reducing activity has not been observed in non-thiol ACEIs, such as enalapril, fosinopril, perindopril, quinapril, and ramipril [62]. However, in vivo studies indicate that both thiol and non-thiol ACEIs enhance the expression of endogenous antioxidant enzymes and improve NO bioavailability, as reflected by increased plasma nitrate and nitrite concentrations [63]. The magnitude of these effects appears to vary among individual agents. Perindopril, for instance, has demonstrated greater anti-inflammatory and antioxidant effects than enalapril, suggesting potential advantages in the management of immune-mediated cardiovascular disorders [64,65,66] (Table 1). These benefits are likely related to ACE inhibition and the resulting vasculoprotective actions of this drug class (Figure 2) [51].

**Table 1:** Effects of antihypertensive drugs and their combinations on nitric oxide, oxidative stress, and inflammation (continues).

Drug	Study Type	Effect on NO	Effect on Oxidative Stress	Effect on Inflammation	Markers Evaluated	Reference
Captopril Epicaptopril Zofenopril Fentiapril	In vitro/acellular	—	↓	—	H_2_O_2_, ^1^O_2_	[57]
Enalapril Perindopril Quinapril Ramipril			N.I.			
Captopril	In vitro/acellular	—	↓	—	TAS	[62]
Enalapril, Fosinopril, Perindopril, Quinapril Ramipril			N.I.			
Captopril Enalapril	In vivo/CF-1 mice	↑	↓	—	GSSG, GSH, Se-GPx, GSSG-Rd, MDA	[63]
Perindopril Enalapril	In vivo/clinical/insulin-resistant patient	—	—	↓↓ ↓	TNF-α, IL-1β and MCP-1, hs-CRP	[64]
Candesartan	In vivo/rats with myocardial fibrosis	—	↓	—	NADPH oxidase-2, ROS, GSH	[67]
Valsartan Amlodipine	In vivo/clinical/patients with end-stage renal disease	↑	↓	—	GSSG, GSH, 8-OHdG, ADMA, SDMA and 13-HODE	[68]
Olmesartan	In vivo/clinical/elderly patients with mild-to-moderate EH	—	↓	↓	SOD, TAS, MDA, AP-1, hs-CRP, ET-1, MCP-1	[69]
Telmisartan	In vivo/clinical/patients receiving epirubicin	—	↓	↓	IL-6, TNF-α, H_2_O_2_, GPx	[70]
Azelnidipine + Olmesartan	In vivo/apolipoprotein E-deficient mice	—	↓	—	O_2_•^−^, NADPH oxidase	[71]
Valsartan Amlodipine	In vivo/clinical/type 2 diabetic patients with hypertension	—	↓	—	Nitrotyrosine	[72]
Olmesartan + azelnidipine	In vivo/clinical/patients with EH	—	↓	↓	hs-CRP, MDA-LDL	[73]
Olmesartan + trichlormethiazide			N.I.	N.I.		
Losartan	In vivo/clinical/healthy men subjected to a simulation of intermittent hypoxia	↑	↓	—	8-OHdG, TAS, nitrotyrosine and end-products of NO metabolism	[74]
Ramipril Losartan	In vivo/clinical/patients with coronary artery disease	↑	↓	—	EC-SOD and FMD	[75]
Verapamil	In vivo/clinical/ hypertensive patients	↑	↓	—	LOOH, MDA, TAS and FBF	[76]
Trandolapril		N.I.				
Verapamil + Trandolapril		↑				
Imidapril L-158.809	In vivo/diabetic rats	—	—	↓	iNOS	[77]
Enalapril	In vivo/Fischer 344 x brown Norway rats	↑	↓	↓	iNOS, eNOS, TNF-α, mTOR and H_2_O_2_	[78]
Perindopril	In vivo/rats with LPS-induced cardiopulmonary inflammation	↑	↓	↓	MDA, NADPH oxidase, GSH, NO_2_^−^, GST, SOD, CAT, NF-kB-p6, MPO, iNOS, eNOS and Akt	[79]
Olmesartan	In vivo/mice with pulmonary hypoxia	—	—	↓	ERK1/2, IL-6, iNOS	[80]
Spironolactone	In vivo/aldosterone-treated rats	—	—	↓	iNOS, IFN-γ, CTGF, MMP-2, TNF-α, CD68, Arg I, and SGK-1 and collagen I	[81]
Nebivolol	In vivo/rats treated with tacrolimus/L-NAME	↑	—	↓	eNOS, iNOS, TGF-β1, TNF-α, IL-1β, collagen 1, NO_2_^−^, urea, creatinine, uric acid, NGAL and KIM-1	[82]
Amlodipine	In vivo/rats with LPS-induced cardiac dysfunction	—	—	↓	TNF-α, iNOS, IκB-α	[83]
Torasemide Furosemide	In vivo/rats with diabetic nephropathy	—	↓	↓	p67phox, HO-1, MCR, VEGF, TNFα, TNFR2	[84]
Trichlormethiazide Chlortalidone	In vivo/clinical/patients with refractory hypertension	—	↓	↓	CRP, 8-isoprostane, MDA-LDL	[85]
Furosemide	In vivo/rats and in vitro/human red blood cells	—	↓	—	Oxygen-radical absorbing capacity	[86]
Eplerenone	In vitro/mouse C2C12 myoblasts	—	↓	—	Intracellular ROS	[87]
Indapamide	In vitro/acellular	—	↓	—	O_2_•^−^ and oxygen-radical absorbing capacity	[88]
5-OH indapamide			↓↓			
Hydrochlorothiazide			N.I.			
Carvedilol	In vivo/clinical/patients with chronic heart failure	—	↓↓	↓	d-ROMs, hs-CRP	[89]
Bisoprolol			↓	↓↓		
Atenolol Labetalol Metoprolol Pindolol Propranolol Sotalol Timolol Carvedilol	In vitro/acellular	—	↓	—	O_2_•^−^, H_2_O_2_, HO•, HOCl, ROO•, NO• and ONOO^−^	[90]
Labetalol	Ex vivo/rabbit neutrophils stimulated with fMLP	—	↓	—	O_2_•^−^,	[91]
Nebivolol	In vitro/H9c2 cells with Ang II–induced mitochondrial dysfunction	—	↓↓	↓↓	Intracellular ROS, NADPH oxidase 2 and 4, CAT, SOD2, MnSOD, GPx, BNIP3, BCL2, BAX, mTORC1, iNOS, TNF-α, NF-κB, MCP-1, TFAM, NRF-1, SIRT3, PGC-1α	[92]
Metoprolol			↓	↓		
Nadolol	In vivo/clinical/normal subjects	—	↓	—	Intracellular ROS, lipid peroxidation	[93]
9-AAP	In vitro/endothelial cells	—	↓↓	—	MDA	[94]
Propranolol			↓			
Propranolol	In vivo/clinical/patients with resistant hypertension	↓	N.I.	—	TAS, NO_3_^−^ and NO_2_^−^	[95]
Atenolol Nebivolol	In vivo/clinical/patients with EH	N.I.	N.I.	—	FMD, MDA, TAS and LOOH	[96]
Nifedipine Amlodipine Telmisartan		N.I.	↓			
Perindopril		↑	↓			
Amlodipine	In vivo/SHRs	↑	↓	—	NO• and ONOO^−^	[97]
Nifedipine	In vivo/clinical/ hypertensive patients	↑	↓	—	FMD, circulating EPCs, MDA-LDL and intracellular ROS	[98]
Nifedipine	In vitro/porcine endothelial cells	↑	↓	—	NO• and O_2_•^−^	[99]
Nifedipine Diltiazem	Ex vivo/human coronary artery endothelial cells	↑	—	—	NO_3_^−^, NO_2_^−^ and eNOS	[100]
Verapamil		N.I.				
Clonidine	In vivo/L-NAME–treated SHRs	↑	↓	—	TAS, MDA, PCO, NO_3_^−^ and NO_2_^−^	[101]
Moxonidine	Ex vivo/RVLM cells from streptozotocin-induced diabetic rats	—	↓	—	HO-1 and CSE	[102]
Moxonidine Clonidine	In vivo/mice with vascular dementia	—	↓	—	MDA, CAT, SOD, GSH	[103]

N.I.: Not identified; ↑: increase; ↓: decrease; ↓↓: greater decrease; 8-OHdG: 8-hydroxy 2-deoxyguanosine; 9-AAP: 9-amino-acridine-propranolol; 13-HODE: 13-hydroxyoctadecadienoic acid; ADMA: asymmetric dimethylarginine; Akt: protein kinase B; AP-1: activator protein-1; Arg I: arginase I; BAX: BCL2-associated X protein; BCL2: B-cell lymphoma 2; BNIP3: BCL2/adenovirus E1B 19 kDa interacting protein 3; CAT: catalase; CD68: cluster of differentiation 68; CSE: cystathionine-γ lyase; CRP: C-reactive protein; CTGF: connective tissue growth factor; d-ROMs: derivatives of reactive oxygen metabolites; EC-SOD: extracellular superoxide dismutase; EH: essential hypertension; eNOS: endothelial nitric oxide synthase; EPCs: endothelial progenitor cells; ERK1/2: extracellular signal-regulated kinases 1 and 2; ET-1: endothelin-1; FBF: Forearm blood flow; FMD: flow-mediated dilation; fMLP: formyl-methionyl-leucyl-phenylalanine; GPx: glutathione peroxidase; GSH: reduced glutathione; GST: glutathione S-transferase; GSSG: glutathione disulfide; GSSG-Rd: glutathione reductase; HO•: hydroxyl radical; HO-1: heme oxygenase-1; HOCl: hypochlorous acid; H_2_O_2_: hydrogen peroxide; hs-CRP: high-sensitivity C-reactive protein; IFN-γ: interferon gamma; IL-1β: interleukin-1 beta; IL-6: interleukin-6; iNOS: inducible nitric oxide synthase; IκB-α: inhibitor of kappaB-alpha; KIM-1: kidney injury molecule-1; L-NAME: Nω-nitro-L-arginine methyl ester; LOOH: lipoperoxides; LPS: lipopolysaccharide; MCR: mineralocorticoid receptor; MDA: malondialdehyde; MDA-LDL: malondialdehyde-modified low-density lipoprotein; MCP-1: monocyte chemoattractant protein-1; MnSOD: manganese SOD; MMP-2: matrix metalloproteinase-2; MPO: myeloperoxidase; mTOR: mammalian target of rapamycin; mTORC1: mammalian target of rapamycin complex 1; NF-kB: nuclear factor kappa B; NRF-1: nuclear respiratory factor 1; NGAL: neutrophil gelatinase-associated lipocalin; NO_2_^−^: nitrite; NO_3_^−^: nitrate; NO•: nitric oxide radical; ONOO^−^: peroxynitrite; PCO: protein carbonyl content; PGC-1α: peroxisome proliferator-activated receptor gamma coactivator 1-alpha; RVLM: rostral ventrolateral medulla; ROS: reactive oxygen species; ROO•: peroxyl radical; SDMA: symmetric dimethylarginine; Se-GPx: selenium-dependent glutathione peroxidase; SGK-1: glucocorticoid kinase-1; SHRs: Spontaneously hypertensive rats; SIRT3: sirtuin 3; SOD: superoxide dismutase; TAS: total antioxidant status; TFAM: mitochondrial transcription factor A; TGF-β1: transforming growth factor-β1; TNF-α: tumor necrosis factor-α; and TNFR2: TNF receptor 2.

### 5.2. Angiotensin II Receptor Blockers

Ang II receptor blockers (ARBs) selectively prevent Ang II from binding to the AT_1_R, thereby counteracting its vascular and metabolic actions. Through this mechanism, ARBs inhibit vasoconstriction, aldosterone, endothelin, and vasopressin release, as well as Ang II-mediated cardiac hypertrophy and oxidative stress [104]. Notable agents within this class include candesartan, irbesartan, losartan, olmesartan, telmisartan, and valsartan [105].

Although both ARBs and ACEIs target the RAAS, they differ in several respects. ACE inhibition influences bradykinin signaling but does not fully suppress Ang II formation, as alternative enzymes such as trypsin, cathepsins, and cardiac chymase can generate Ang II. While the contribution of these pathways to hypertension remains uncertain, AT_1_R blockade by ARBs effectively neutralizes the effects of Ang II regardless of its enzymatic source [106].

Several ARBs have demonstrated antioxidant properties beyond BP reduction, including candesartan [67], valsartan [68], Olmesartan [69], and telmisartan [70]. Evidence suggests that combination therapy with two antihypertensive agents not only enhances BP control but also reduces oxidative stress [71]. For example, the combination of valsartan and amlodipine demonstrated greater antioxidant efficacy than either drug alone [72]. Comparable results have been reported for other combinations, such as olmesartan with azelnidipine (Table 1) [73].

Blockade of AT_1_R by losartan has been shown to effectively reduce oxidative stress and limit peroxynitrite formation induced by intermittent hypoxia [74]. Moreover, losartan treatment improved endothelial function, accompanied by increased NO bioavailability and enhanced activity of extracellular SOD (EC-SOD), the principal antioxidant enzyme in the human arterial wall (Figure 2) [75].

In patients with metabolic syndrome and endothelial dysfunction, irbesartan therapy significantly improved flow-mediated dilation (FMD) and reduced plasma concentrations of interleukin-6, plasminogen activator inhibitor-1, and 8-isoprostane—biomarkers of inflammation, atherothrombotic activity, and oxidative stress, respectively [76].

Reductions in inflammatory markers may also be associated with decreased inducible NOS (iNOS) activity, as suppression of RAAS signaling attenuates lipopolysaccharide (LPS)-induced iNOS activation [77]. Several drug classes, including ACEIs [78,79], ARBs [80], diuretics [81], β-blockers [82], and calcium channel blockers [83], have been shown to downregulate iNOS expression under various conditions. In macrophages, iNOS expression is regulated by NF-κB, whose activation is promoted by ROS. Antihypertensive agents with antioxidant properties inhibit this pathway, thereby limiting inflammatory signaling (Table 1; Figure 2) [107].

### 5.3. Diuretics

Diuretics reduce BP by decreasing renal sodium and water reabsorption, which enhances diuresis and lowers blood volume and cardiac preload, ultimately leading to reduced systemic arterial pressure. These agents are classified based on their site of action along the nephron [108]. Currently, four major classes of diuretics are commonly used in the management of AH: thiazide and thiazide-like diuretics, which inhibit the Na^+^/Cl^−^ cotransporter in the distal convoluted tubule; potassium-sparing diuretics, which act on the distal tubule membrane by blocking sodium reabsorption in exchange for potassium; aldosterone antagonists, which compete with aldosterone at the mineralocorticoid receptor; and loop diuretics, which inhibit the Na^+^/K^+^/2Cl^−^ cotransporter in the thick ascending limb of the loop of Henle [109].

Commonly prescribed diuretics include amiloride, chlorthalidone, eplerenone, furosemide, hydrochlorothiazide, spironolactone, triamterene, indapamide, torsemide, and metolazone [110]. In in vivo studies, torsemide and furosemide reduced the expression of the p67phox subunit, a marker associated with oxidative stress. Notably, torsemide exerted more pronounced effects, including downregulation of the mineralocorticoid receptor and increased mRNA expression of heme oxygenase-1 (HO-1), an enzyme recognized for its anti-inflammatory and antioxidant properties (Table 1) [84].

In experimental models of renovascular hypertension, hydrochlorothiazide and spironolactone demonstrated antioxidant effects and reduced matrix metalloproteinase-2 (MMP-2) activity, thereby attenuating hypertension-associated vascular remodeling [14]. Chlorthalidone has also exhibited antioxidant activity in patients with hypertension refractory to combined therapy with calcium channel blockers (CCBs) and ARBs. In addition to improving BP control, chlorthalidone significantly reduced lipid peroxidation levels [85].

In vitro studies showed that furosemide [86], eplerenone [87], indapamide, and 5-OH indapamide also exhibit antioxidant activity, with 5-OH indapamide demonstrating greater efficacy than vitamin E [88]. However, the precise molecular mechanisms underlying these effects remain incompletely elucidated. Other diuretics, such as metolazone, amiloride, and triamterene, still lack experimental investigations evaluating their antioxidant potential. No evidence of antioxidant activity has been reported for chlorothiazide, although it is a more potent structural analogue of hydrochlorothiazide. In contrast, the potassium-sparing diuretic spironolactone has been shown to attenuate NADPH oxidase activity and oxidative stress in aortic vascular smooth muscle cells (Figure 2, Table 1) [14].

### 5.4. Beta-Blockers

Beta-blockers act on β-adrenergic receptors, blocking the action of endogenous catecholamines that increase myocardial contractility, heart rate, stroke volume, and, consequently, BP. These drugs are classified according to their selectivity for β-receptor subtypes [111]. Cardioselective agents such as atenolol, bisoprolol, metoprolol, and nebivolol exhibit greater affinity for β_1_ receptors, which are predominantly expressed in the myocardium and kidneys. In contrast, non-selective agents such as carvedilol, labetalol, nadolol, and propranolol act on both β_1_ and β_2_ receptors, the latter being widely distributed in the lungs, liver, brain, kidneys, and heart. In addition, carvedilol and labetalol also block α_1_-adrenergic receptors located in vascular smooth muscle, conferring an additional vasodilatory effect [112].

Bisoprolol and carvedilol has been shown to reduce oxidative stress and levels of inflammatory markers in patients with chronic heart failure. Both drugs decreased serum concentrations of high-sensitivity C-reactive protein (hsCRP), an inflammatory marker, and d-ROMs (derivatives of ROS), a marker of oxidative stress (Table 1) [89].

In vitro studies showed that the β-blockers atenolol, labetalol, metoprolol, pindolol, propranolol, sotalol, timolol, and carvedilol have no capacity to scavenge the superoxide radical. Only labetalol and timolol were effective in eliminating hydrogen peroxide. Labetalol and pindolol showed the highest efficiency in scavenging the hydroxyl radical (•OH), followed by propranolol, sotalol, timolol, atenolol, and metoprolol, respectively. Moreover, all compounds tested were effective in eliminating peroxynitrite, except for timolol and labetalol [90]. In contrast, in vivo studies showed that labetalol dose-dependently inhibits superoxide generation, suggesting a possible indirect modulation of oxidative stress [91].

In a recent study, Gul et al. (2025) [92] investigated the effects of metoprolol and nebivolol on Ang II-induced mitochondrial impairment using H9c2 cardiomyocytes. Metoprolol increased the expression of the antioxidant enzyme manganese SOD (MnSOD), whereas nebivolol upregulated MnSOD, SOD-2, and glutathione peroxidase (GPx). Both drugs reduced pro-apoptotic markers, with nebivolol showing greater efficacy. Furthermore, inflammatory and hypertrophic markers were also attenuated by both agents.

In healthy individuals receiving nadolol, a reduction in leukocyte-derived ROS generation and a significant decrease in linoleic acid oxidation were observed, suggesting a systemic antioxidant effect [93]. In endothelial cells, propranolol exhibited a protective effect against acute GSH depletion and demonstrated significant cytoprotective activity against free radical-mediated loss of cell viability [94]. However, in patients with resistant hypertension, propranolol did not show a significant effect on the balance between oxidative stress and antioxidant defenses (Figure 2, Table 1) [95].

### 5.5. Calcium Channel Blockers

Calcium channel blockers (CCBs) exert their therapeutic effects by inhibiting voltage-dependent L-type calcium channels located in the myocardium and vascular smooth muscle. In the heart, this inhibition reduces calcium influx into nodal cells, suppressing the propagation of action potentials in the sinoatrial and atrioventricular nodes, resulting in negative inotropy and heart rate control. In vascular smooth muscle, blockade of these channels promotes arteriolar relaxation, reducing sympathetic tone and peripheral vascular resistance, ultimately leading to decreased BP and antiarrhythmic effects [104].

CCBs are classified into two major groups based on their chemical structure: dihydropyridines, represented by nifedipine, felodipine, and amlodipine, which have greater affinity for vascular smooth muscle; and non-dihydropyridines, such as verapamil and diltiazem, which exhibit greater cardiac selectivity. These structural differences determine not only pharmacodynamic specificity but also additional properties. Dihydropyridines, for example, are capable of antagonizing the mineralocorticoid receptor, an effect not observed with non-dihydropyridine CCBs (Table 1) [105].

Nifedipine and amlodipine, but not diltiazem, have been shown to attenuate endothelial dysfunction, leukocyte activation, and oxidative stress independent of their antihypertensive actions, an effect associated with the possible blockade of dihydropyridine-sensitive calcium channels expressed in leukocytes [106]. Amlodipine has also been shown to reduce oxidation of lipids, thiols, proteins, and nucleic acids, in addition to decreasing the formation of ADMA [68,96] and peroxynitrite [97], both recognized markers of endothelial dysfunction.

In patients with essential hypertension, treatment with nifedipine for four weeks increased FMD, reduced oxidative stress, and suppressed apoptosis of circulating endothelial progenitor cells, thereby contributing to the preservation of endothelial integrity [98]. Beyond its antioxidant and antiapoptotic effects, nifedipine and diltiazem also enhanced NO bioavailability through upregulation of eNOS expression [99,100] and reduction of peroxynitrite formation [97].

Antioxidant effects have also been observed with verapamil, which significantly improved endothelium-dependent vasodilation and reduced mean peripheral vascular resistance in patients with essential hypertension. These findings were attributed to increased total plasma antioxidant capacity and reduced levels of malondialdehyde (MDA) (Figure 2, Table 1) [76].

### 5.6. Centrally Acting Antihypertensive Agents

Centrally acting antihypertensive drugs act predominantly by inhibiting central sympathetic activity, exerting agonist effects on α_2_-adrenergic receptors and/or type I_1_ imidazoline receptors. Presynaptic α_2_ receptors, expressed in adrenergic neurons, are coupled to inhibitory G proteins (Gi), whose activation reduces the release of noradrenergic neurotransmitters, resulting in decreased plasma catecholamine levels and, consequently, attenuation of central sympathetic outflow directed to the heart and blood vessels. Similarly, agonism of I_1_ receptors also promotes a reduction in sympathetic activity, although the molecular mechanisms involved have not yet been fully elucidated [109,113].

Clonidine and lofexidine act as mixed agonists of α_2_-adrenergic and I_1_ imidazoline receptors. Although effective in reducing BP, these agents have been used less frequently in clinical practice due to the availability of safer therapeutic options with fewer adverse effects, and are currently employed mainly for other clinical indications, such as the management of opioid withdrawal and control of vasomotor symptoms. In an experimental animal model of hypertension, clonidine was shown to reduce oxidative stress and increase total antioxidant capacity in cardiac tissue [101]. Moxonidine and rilmenidine, in turn, exhibit greater selectivity for I_1_ receptors, with a lower incidence of central adverse effects, such as sedation and drowsiness, typically observed in agonists with predominant affinity for α_2_-adrenergic receptors. In addition to their antihypertensive actions, both drugs have also demonstrated antioxidant properties, although the mechanisms underlying these effects remain incompletely understood (Figure 2, Table 1) [102,103,114].

Overall, centrally acting antihypertensive agents—similar to drugs from other classes previously described—exhibit experimental and clinical evidence that is at times divergent. Variability among findings may be attributed to methodological differences, including the animal models employed, methods of oxidative stress induction, cell lines used, associated comorbidities, and distinct techniques for quantifying reactive species and oxidation products. These inconsistencies underscore the need for further studies, particularly those integrating in vitro and in vivo findings, to clarify the molecular mechanisms responsible for the antioxidant and CV effects of these pharmacological agents.

## 6. Antioxidant and Vascular Effects of a Nitrate- and Nitrite-Rich Diet

The intake of foods rich in inorganic nitrate and nitrite represents an alternative and complementary pathway to the endogenous production of NO mediated by eNOS. Upon entering the nitrate–nitrite–NO pathway, nitrate is converted into nitrite by the oral microbiota and subsequently reduced to NO in the acidic gastric environment or under conditions of low oxygen tension [115,116,117]. This eNOS-independent route is particularly relevant in pathological states characterized by endothelial dysfunction and oxidative stress, such as hypertension, in which NO bioavailability is compromised [31,118].

Multiple studies have reported the effects of nitrate-rich diets on BP, demonstrating increased plasma and tissue concentrations of NO metabolites associated with significant reductions in BP levels [119]. However, beyond their effects on BP, nitrate and nitrite also possess antioxidant properties [18]. Dietary nitrate increased mitochondrial ATP synthesis capacity and reduced mitochondrial proton leak by decreasing the expression of the ATP/ADP translocase, a protein involved in proton conductance [120]. Nitrate intake prior to exercise also increased the expression of peroxisome proliferator-activated receptor gamma coactivator 1-alpha (PGC-1α) and mitofusin 2 (Mfn2), proteins involved in mitochondrial biogenesis and remodeling [121]. Other studies attribute to nitrate the ability to reduce mitochondrial hydrogen peroxide release [122] and to lower oxygen consumption during exercise [123]. Similar results were observed with nitrite treatment, which decreased mtROS release, increased NO bioavailability, and improved endothelial function impaired by aging [124]. However, despite evidence suggesting that nitrate and nitrite intake improves mitochondrial function, these findings need to be validated in clinical studies involving hypertensive patients.

According to a study conducted by Fejes et al., daily consumption of nitrate-rich beetroot juice significantly reduced oxidative stress in hypertensive adults compared with low-nitrate juice, suggesting that, despite the presence of other bioactive compounds, nitrate content was the primary determinant of the antioxidant effect [125,126]. It is possible that the composition of beetroot, as well as that of other nitrate-rich vegetables, confers benefits to cardiovascular health. The phytocomplex, comprising vitamins, minerals, and phytochemicals, may explain, at least in part, why nitrate and nitrite derived from vegetables are not associated with the development of malignant neoplasms, in contrast to what is observed when these anions are used as preservatives in processed meats [127,128].

Nitrate and nitrite exert marked antioxidant effects, acting at multiple levels of redox regulation [129]. One of the primary mechanisms involves the inhibition of NADPH oxidase, a key enzyme responsible for generating the superoxide anion [130]. Reduced NADPH oxidase activity decreases the formation of peroxynitrite, thereby preserving endothelial signaling and preventing eNOS uncoupling, which is generally induced by BH_4_ oxidation [131].

Another key mechanism involves activation of the Nrf2 pathway, a central transcriptional regulator of antioxidant defense, a key transcriptional regulator of cellular defenses against oxidative stress. Our previous findings demonstrated that increased nitrite intake promoted nuclear accumulation of Nrf2 and upregulation of mRNA expression of Nrf2-regulated antioxidant genes, including SOD-1, catalase (CAT), GPx, thioredoxin-1 (TRDX-1), and thioredoxin-2 (TRDX-2) [17].

The increase in NO bioavailability induced by nitrate and nitrite intake also facilitates S-nitrosylation of cysteine residues in proteins involved in diverse signaling pathways [132,133,134], including kinases mediating Ang II-related responses [135]. S-nitrosylation of Kelch-like ECH-associated protein 1 (Keap1), which is normally complexed with Nrf2 in the cytoplasm, promotes its dissociation and subsequent nuclear translocation of Nrf2. Once in the nucleus, Nrf2 binds to antioxidant response elements (AREs), inducing the expression of protective genes such as HO-1, NAD(P)H quinone oxidoreductase-1 (NQO1), and additional antioxidant enzymes [136,137]. Activation of these pathways results in increased total antioxidant capacity and improved regulation of vascular redox homeostasis [125,138,139]. Furthermore, regular consumption of nitrate-rich foods is associated with increased levels of reduced GSH and an improved GSH/GSSG ratio, suggesting strengthening of the non-enzymatic antioxidant system [125].

Collectively, these mechanisms culminate in significant improvements in endothelial function, increased FMD, and reduced arterial stiffness, effects observed in both normotensive and hypertensive individuals [140,141]. The integration between the nitrate–nitrite–NO pathway and activation of the Nrf2/HO-1/NQO1 axis [137] reinforces the multifactorial role of nitrate-rich diets as an adjuvant strategy to antihypertensive therapy, particularly in pharmacological regimens lacking direct antioxidant activity.

## 7. Contribution of a Nitrate- and Nitrite-Rich Diet to the Treatment with Antihypertensive Drugs Lacking Antioxidant Effects

AH is a condition that involves not only hemodynamic dysfunction but also redox imbalance and vascular inflammation. Although pharmacological therapy is essential for BP control, consistent evidence regarding the antioxidant potential of many drugs used for this purpose is still lacking. When unable to attenuate oxidative stress, antihypertensive therapy may not be fully effective, as it neither prevents the oxidative damage underlying hypertension nor adequately restores NO bioavailability [142]. Consequently, even when BP is reduced to suboptimal or optimal levels, a substantial residual cardiovascular risk may persist [143,144].

**Figure 3:**
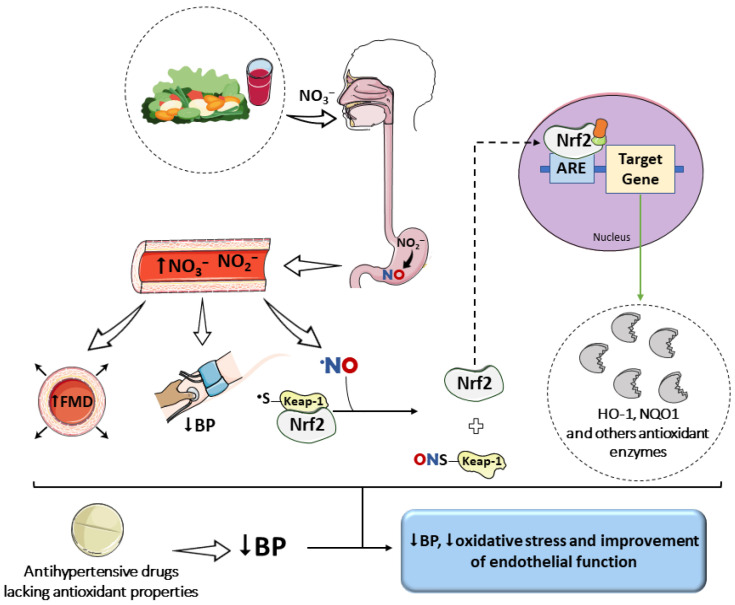
Impact of a nitrate-rich diet on oxidative stress and on the effects of antihypertensive drugs. The increase in NO bioavailability, evidenced by elevated plasma and tissue levels of nitrate and nitrite, favors S-nitrosylation reactions. This post-translational modification of the Keap1 protein promotes the release of Nrf2 and the upregulation of antioxidant enzyme gene expression. This effect, combined with the vasodilatory action of NO, may complement the benefits of drugs that reduce blood pressure (BP) but lack inherent antioxidant properties. Parts of the figure were drawn using pictures from Servier Medical Art. Servier Medical Art by Servier is licensed under a Creative Commons Attribution 4.0 unported license.

Some clinical studies have demonstrated that antioxidant supplementation does not significantly reduce cardiovascular risk [145], whereas higher dietary intake of antioxidants has been consistently associated with cardioprotective effects [146,147,148]. In this context, a nitrate-rich diet—characteristic of dietary patterns with high vegetable intake—emerges as a promising adjuvant strategy capable of complementing conventional antihypertensive therapy [149]. Clinical studies demonstrate that supplementation with beetroot juice, one of the most concentrated dietary sources of nitrate, enhances the antihypertensive effect of routinely used medications. In pharmacologically treated patients, daily intake of nitrate-rich beetroot juice for 4 weeks resulted in additional reductions of approximately 8 mmHg in systolic BP, along with significant increases in FMD (Figure 3) [150,151].

Beyond interventions involving beetroot juice, observational studies on vegetarian and vegan diets—naturally rich in nitrate and antioxidants—have shown lower arterial stiffness and reduced BP levels compared with omnivorous diets [152]. Among hypertensive individuals taking losartan, adherence to a Dietary Approaches to Stop Hypertension (DASH) diet, characterized by high consumption of leafy vegetables and dietary nitrate, was associated with improved BP control [153]. These effects may be attributed not only to nitrate intake but also to the intrinsic antioxidant properties of this dietary pattern, similarly observed in Mediterranean-based diets [154,155].

Although evidence suggests that high nitrate intake enhances responses to pharmacological therapy [150,151,156], it remains unclear which classes of antihypertensive drugs benefit most from this interaction. Considering the extensive literature demonstrating the central role of oxidative stress in vascular function and NO bioavailability [32], as well as the benefits of mitigating this redox imbalance for BP control and CV risk reduction [157], it is plausible to propose that dietary supplementation with nitrate and nitrite may be particularly advantageous in therapeutic regimens lacking intrinsic antioxidant properties. Such benefits arise not only from the antioxidant actions of these anions but also from their direct effects on endothelial function and NO bioavailability [124].

According to Kapil et al. [150], hypertensive patients receiving antihypertensive therapy benefited from the consumption of nitrate-rich beetroot juice, which reduced pulse wave velocity (PWV) by 0.59 m/s and significantly increased FMD by approximately 20%. PWV is considered the gold standard for assessing arterial stiffness and is a well-established predictor of cardiovascular events. In the same study, the placebo (nitrate-depleted beetroot juice) did not induce significant changes in functional parameters, further demonstrating that the cardioprotective effects of beetroot juice are dependent on inorganic nitrate.

In addition to its effects on endothelial function, combining antihypertensive drugs with a nitrate- and nitrite-rich diet may allow the use of lower pharmacological doses, although its impact on residual cardiovascular risk has not yet been fully established. If confirmed, this could represent a clinically meaningful advancement, as it could reduce the occurrence of adverse events associated with conventional therapy. Future studies should investigate whether nitrate-rich diets can enhance BP control with a lower medication burden, particularly in elderly patients and those on polypharmacy, in whom reduced pharmacological exposure provides additional clinical and safety benefits [158,159]. Moreover, the sustained improvement in endothelial function observed with such combinations may help mitigate CV complications associated with the chronic nature of hypertension [160].

## 8. Future Perspectives

This narrative review integrates current evidence on dietary inorganic nitrate and nitrite intake with the pharmacodynamics of antihypertensive drugs, focusing on redox imbalance as a determinant of therapeutic efficacy. In contrast to reviews that address dietary nitrate or antihypertensive pharmacotherapy separately, this work examines the role of nitrate- and antioxidant-rich diets in the context of antihypertensive agents that lack intrinsic redox-modulating properties. Moreover, it presents a mechanistic framework linking nitrate and nitrite intake to Nrf2/HO-1/NQO1 signaling, mitochondrial function, and NO bioavailability, offering an integrated perspective on vascular effects beyond BP reduction.

Available evidence suggests that the combination of antihypertensive pharmacotherapy and nitrate-rich dietary interventions is associated with modulation of oxidative stress, endothelial dysfunction, and NO signaling, with consequent benefits for BP control and cardiovascular risk reduction. There is a clear need for long-term randomized clinical trials to define class-specific interactions between antihypertensive drugs and dietary nitrate intake. The findings of this review support the incorporation of nitrate-rich vegetables into dietary guidelines as an adjunct strategy to conventional antihypertensive therapy.

## Data Availability

No new data were created or analyzed in this study.
